# Genomic and phenotypic characterization of in vitro-generated *Chlamydia trachomatis* recombinants

**DOI:** 10.1186/1471-2180-13-142

**Published:** 2013-06-20

**Authors:** Brendan M Jeffrey, Robert J Suchland, Steven G Eriksen, Kelsi M Sandoz, Daniel D Rockey

**Affiliations:** 1 Molecular and Cellular Biology Program, Oregon State University, Corvallis, OR, USA; 2 Department of Biomedical Sciences, Oregon State University, Corvallis, OR, USA; 3 Division of Allergy and Infectious Disease, Department of Medicine, University of Washington, Seattle, WA, USA

**Keywords:** Chlamydia, Recombination, Hotspot, Attachment, Secondary inclusions

## Abstract

**Background:**

Pre-genomic and post-genomic studies demonstrate that chlamydiae actively recombine in vitro and in vivo, although the molecular and cellular biology of this process is not well understood. In this study, we determined the genome sequence of twelve *Chlamydia trachomatis* recombinants that were generated in vitro under antibiotic selection. These strains were used to explore the process of recombination in *Chlamydia spp.*, including analysis of candidate recombination hotspots, and to correlate known *C. trachomatis* in vitro phenotypes with parental phenotypes and genotypes.

**Results:**

Each of the 190 examined recombination events was the product of homologous recombination, and no candidate targeting motifs were identified at recombination sites. There was a single deletion event in one recombinant progeny that resulted in the removal of 17.1 kilobases between two rRNA operons. There was no evidence for preference for any specific region of the chromosome for recombination, and analyses of a total of over 200 individual recombination events do not provide any support for recombination hotspots in vitro. Two measurable phenotypes were analyzed in these studies. First, the efficiency of attachment to host cells in the absence of centrifugation was examined, and this property segregated to regions of the chromosome that carry the polymorphic membrane protein (Pmp) genes. Second, the formation of secondary inclusions within cells varied among recombinant progeny, but this did not cleanly segregate to specific regions of the chromosome.

**Conclusions:**

These experiments examined the process of recombination in *C. trachomatis* and identified tools that can be used to associate phenotype with genotype in recombinant progeny. There were no data supporting the hypothesis that particular nucleotide sequences are preferentially used for recombination in vitro. Selected phenotypes can be segregated by analysis of recombination, and this technology may be useful in preliminary analysis of the relationship of genetic variation to phenotypic variation in the chlamydiae.

## Background

*Chlamydia trachomatis* is a Gram-negative obligate intracellular bacterium that is a leading cause of preventable blindness and sexually transmitted diseases worldwide [1]. Much of the biology of infection and disease remains unclear in this system, owing largely to the lack of a routine genetic system for these organisms. While many aspects of these challenges have recently been overcome [2,3], the use of genetic transformation in this system is just beginning to be exploited. One aspect of chlamydial biology that is poorly understood involves the mechanism of lateral gene transfer among chlamydial strains both in the laboratory and, most likely, in patients. Coinfection of host cells in vitro with chlamydial isolates encoding different drug resistance markers lead to generation of dual resistant recombinant progeny [4,5]. These results lend support to pre- and post-genomic analyses demonstrating that chlamydiae recombine in infected hosts [6-12], likely following infection of a patient by multiple strains [13]. *Chlamydia* spp. encode no recognizable bacterial gene transfer systems, thus the mechanisms underlying chlamydial recombination remain unknown.

*C. trachomatis* and many other chlamydiae are differentiated into distinct serovars based on antibody specificity to the major outer membrane protein (MOMP or OmpA), encoded by *ompA*. Serovars and subserovars of *C. trachomatis* fall into three groups those associated with trachoma (serovars A, B, and C), those associated with non-invasive sexually transmitted infections of the urogenital tract (serovars D through K), and those associated with invasive lymphogranuloma (LGV; serovars L1 to L3) [14]. This historical classification system has recently been modified to a genotypic characterization of strains, both by sequencing of *ompA* and the inclusion of a variety of other markers in the analysis [15-17]. Nevertheless, many of the biological differences among chlamydiae still can be grouped by the serovar-based classification scheme. Clinically relevant differences among the chlamydiae include host tropism, variation in disease outcome, and in vitro biology. With some exceptions (reviewed in [18]), such as tryptophan utilization [19,20] and fusogenicity of inclusions [21], the relationship between genotype and phenotype is not clear in vitro and certainly not with regards to how the phenotypes observed in cell culture relate to the disease potential of a particular strain. Two such phenotypes that are different among *C. trachomatis* strains include the historical difference among serovars regarding attachment and invasion in the presence or absence of centrifugation during the infectious process [22], and secondary inclusion formation by different chlamydial strains [23]. Deciphering the genetic basis of these and other phenotypes is complicated by the relatively primitive molecular genetic techniques that have been available for studying chlamydial biology, although this situation is changing.

In the present study, genetically mosaic recombinant strains from parents with differing cell culture phenotypes were generated in vitro, cloned by limiting dilution, and subjected to complete genome sequence analysis. These strains, the parentals used in the crosses, and selected clinical isolates were used to investigate the process of chlamydial genetic exchange, and to develop and test a system for a primary examination of attachment and invasion as well as secondary inclusion formation phenotypes in *C. trachomatis*.

## Results

### Generation of recombinant strains

**Figure 1:**
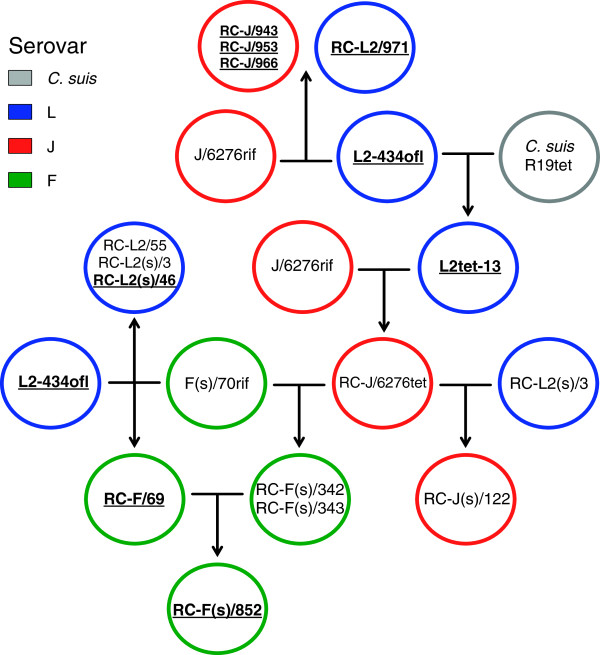
**The genealogy of recombinant strains generated and explored in this study.** The figure shows the parental strains used to generate recombinant strains. In each case, the parents in a cross are connected by a horizontal line, and the progeny are at the end of the arrow. In some cases, the progeny of one cross was used as a parent in a subsequent cross. Primary parental strain names includes drug resistance, and all recombinant strains (indicated by prefix RC- ) are both rifampicin and ofloxacin resistant. The colors used indicate the OmpA phenotype of each strain, as determined by fluorescence microscopy and genome sequence analysis. Strains containing the plasmid are shown in bold face and underlined. Crosses involving three parents are not shown because no triply drug resistant strains could be recovered.

**Figure 2:**
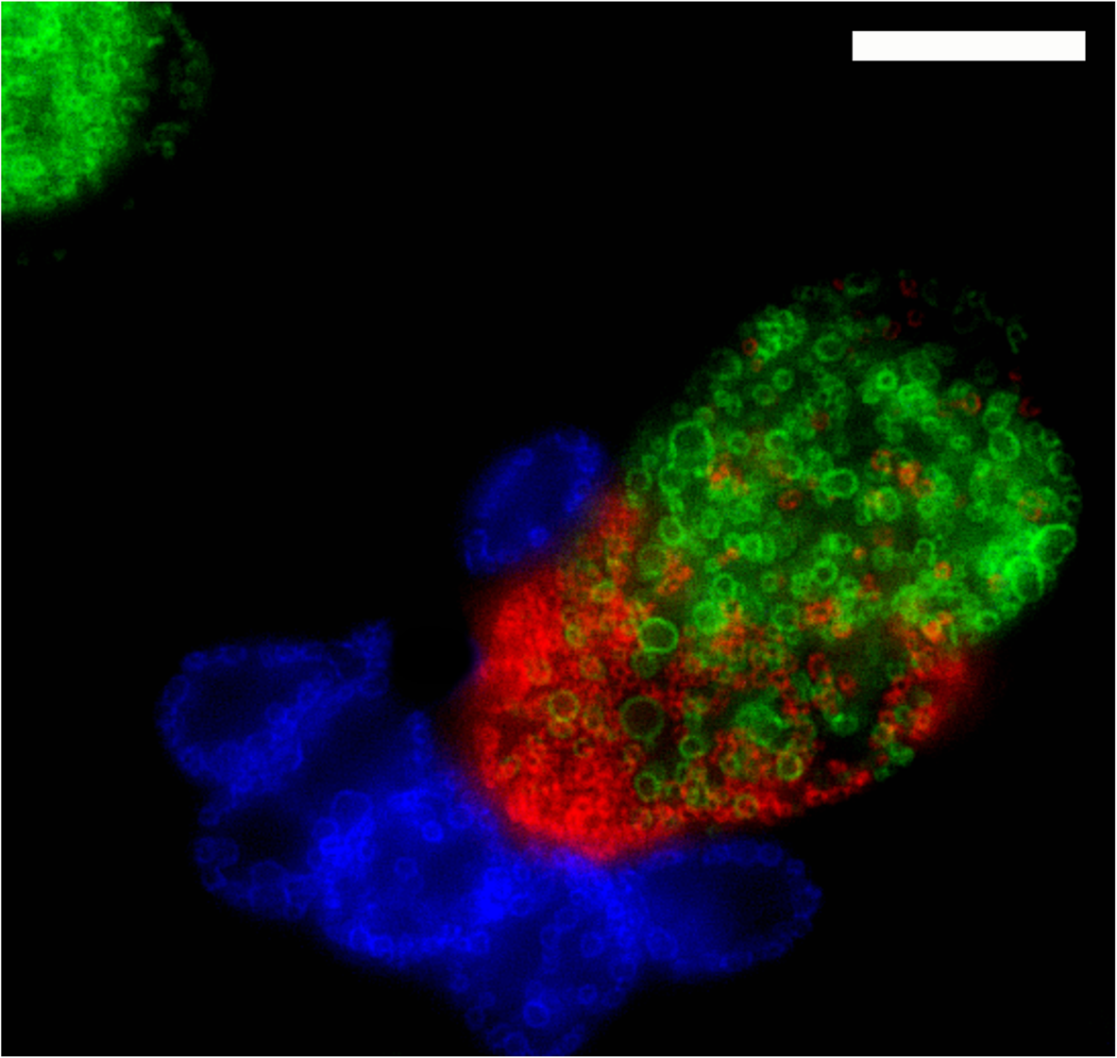
**Fluorescent microscopy showing host cells infected with three *C. trachomatis *strains.** Strains were labeled with primary antibodies against OmpA. Cells are infected with L2-434 (green), J/6276 (red), and the inclusion fusion negative strain F(s)/70 (blue). Scale bar, 5 μm.

A collection of recombinant strains was generated using parent strains within serovars J, F, and L2 (Table 1, Figure 1). These included IncA-positive strains J/6276 and L2-434, and the IncA negative strain F(s)/70. In some cases, crosses involved two parents (i.e. crosses 1–6, 11,12); while in other cases three-way crosses were attempted (i.e. Table 1, crosses 7–10). Each of these crosses yielded expected inclusion fusion patterns, leading to cells infected with both IncA-positive strains forming fused inclusions, while cells infected with either IncA-positive strain and the F(s)/70 IncA-negative strain having multiple inclusions per cell. Mixtures of all three of these strains led to cells having mixed inclusions containing the two IncA-positive strains, and a set of isolated inclusions containing the IncA-negative F(s)/70 strain (Figure 2). While progeny with each possible combination of two antibiotic resistance markers were routinely identified in the three-way crosses, no triply resistant strain was recovered in any experiment. Additionally, no single progeny strain had sequence at any informative position from each of the three parents in a three-way cross. Recombinant progeny were generated in crosses of both IncA-positive and IncA-negative parents, with no apparent difference in the rate of recovery of recombinants relative to the IncA status of the parent (not shown).

**Table 1:** Phenotypes of parents and progeny in recombinant crosses

				**MIC (μg/ml)**				**Phenotype**		
**Cross**	**Progeny**	**Parental strains**	**OmpA**	**Rif**	**Ofl**	**Tet**	**Plasmid**	**Inclusion fusion**	**Attachment**	**2° inclusion formation**
1	**RC-J/6276tet**		J	8	0.5	8	-	+	-	2
		L2-434tet-13	L2	0.008	16	8	+	+	+	1
		J/6276rif	J	8	0.5	0.032	-	+	-	1
2	RC-F(s)/342		F	32	0.5	8	-	-	-	N/A
		**RC-J/6276tet-rif**	J	8	0.5	8	-	+	-	1
		F(s)/70rif	F	32	0.5	0.032	-	-	-	N/A
3	RC-L2(s)/46		L2	32	16	0.032	+	-	+	N/A
		L2-434ofl	L2	0.008	16	0.032	+	+	+	1
		F(s)70rif	F	32	0.5	0.032	-	-	-	N/A
4	**RC-F/69**		F	32	4	0.032	+	+	+	1
		L2-434ofl	L2	0.008	16	0.032	+	+	+	1
		F(s)70rif	F	32	0.5	0.032	-	-	-	N/A
5	**RC-L2(s)/3**		L2	32	4	0.032	-	-	+	N/A
		L2-434ofl	L2	0.008	16	0.032	+	+	+	1
		F(s)70rif	F	32	0.5	0.032	-	-	-	N/A
6	RC-L2/55		L2	32	4	0.032	-	+	+	N/A
		L2-434ofl	L2	0.008	16	0.032	+	+	+	1
		F(s)/70rif	F	32	0.5	0.032	-	-	-	N/A
7	RC-J/953		J	8	16	0.032	+	+	+	4
		L2-434ofl	L2	0.008	16	0.032	+	+	+	1
		**RC-F(s)/343tet-rif**	F	32	0.5	8	-	-	-	N/A
		J/6276rifR	J	8	0.5	0.032	-	+	-	1
8	RC-J/943		J	8	16	0.032	+	+	+	1
		L2-434/ofl	L2	0.008	16	0.032	+	+	+	1
		**RC-F(s)/343tet-rif**	F	32	0.5	8	-	-	-	N/A
		J/6276rif	J	8	0.05	0.032	-	+	-	1
9	RC-J/966		J	8	16	0.032	+	+	+	1
		L2-434/ofl	L2	0.008	16	0.032	+	+	+	1
		**RC-F(s)/343tet-rif**	F	32	0.5	8	-	-	-	N/A
		J/6276rif	J	8	0.5	0.032	-	+	-	1
10	RC-L2/971		J	8	16	0.032	+	+	+	4
		L2-434/ofl	L2	0.008	16	0.032	+	+	+	1
		**RC-F(s)/343tet-rif**	F	32	0.5	8	-	-	-	N/A
		J/6276rif	J	8	0.5	0.032	-	+	-	1
11	RC-F(s)/852		F	32	4	8	+	-	-	N/A
		**RC-F/69**	F	32	4	0.032	+	+	+	1
		**RC-F(s)/343tet-rif**	F	32	0.5	8	-	-	-	N/A
12	RC-J(s)/122		J	32	4	8	-	-	+	N/A
		**RC-L2(s)/3**	L2	32	4	0.032	-	-	+	N/A
		**RC-J/6276tet-rif**	J	8	0.5	8	-	+	-	1

Plasmid phenotype tests for presence and genotype of plasmid.

Progeny strains used in subsequent recombinant crosses are shown in bold face.

### Genome sequence analysis of recombinant strains

**Figure 3:**
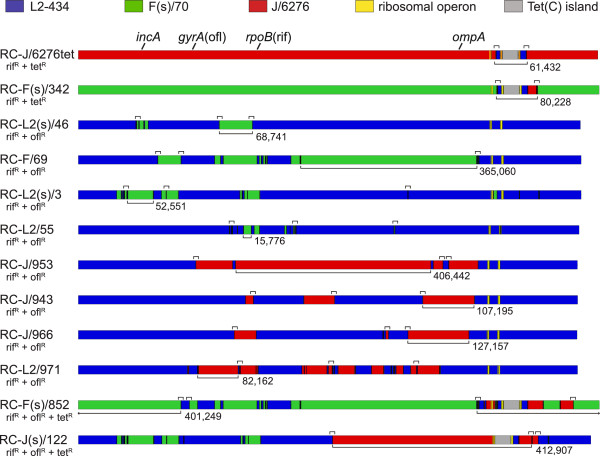
**Genome maps of recombinant strains, derived from complete nucleotide sequence analysis.** The colors used in recombinant maps indicate the parental genotype, as is indicated at the top of the figure. The Tet(C) island is originally from *C. suis* R19. The approximate location of the genetic markers used in the construction of the recombinant genomes is shown above the RC-J/6276tet genome map. Below each strain name is the antibiotic resistance markers that the recombinant strain carries. The bracket and number below each genome map indicate the largest size of contiguous integrated DNA. The small brackets above each genome map indicate crossover regions that were confirmed by PCR amplification and Sanger sequencing.

The genomes of the twelve recombinant strains were sequenced using Illumina paired-end technology (Figure 3). In all recombinant strains, the sequences surrounding the individual resistance markers were derived from the appropriate parent, supporting the conclusion that these were recombinant strains and not spontaneous mutants that emerged during the selection process. There was evidence of a single random mutation in one recombinant, strain RC-L2(s)/3. This mutation was a G (L2-434 sequence) to A [RC-L2(s)/3] substitution at position 293,505 (genome accession CP002676), resulting in an alanine to valine amino acid change in the protein product of CT258. This same mutation was identified in the RC-J(s)/122 genome, a progeny of a cross in which RC-L2(s)/3 was a parent. There was no other evidence of random base change in any other sequenced recombinant genome.

**Figure 4:**
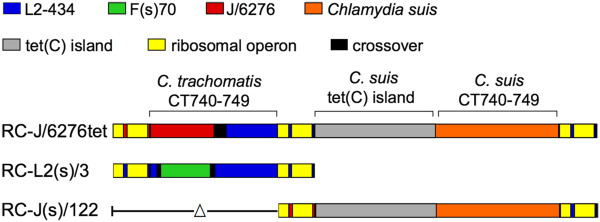
**Schematic diagram of the CT740 to CT749 regions in selected recombinant sequences.** The colors used indicate the genotype of a given region. The ribosomal operons are shown in yellow, and crossover sites are shown in black. The deletion of the *C. trachomatis* homologous region of CT740 to CT749 in the RC-J(s)/122 sequence is indicated by the delta symbol.

**Figure 5:**
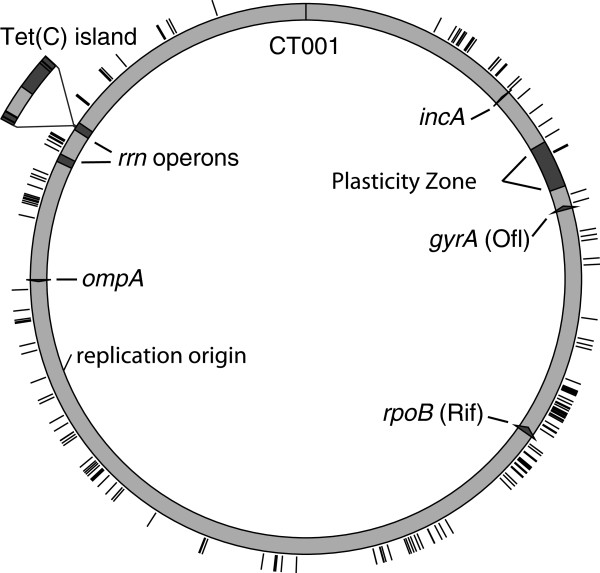
**The genomic location of crossover regions in each of the twelve sequenced recombinant progeny strains.** The sequenced strain D/UW3Cx gene designations were used as the reference, with the location of gene CT001 indicated at the top of representative genome. The black tick marks indicates the location of a crossover region. The location of the plasticity zone is shown in gray.

With one exception, the exchange of DNA in each recombination event yielded products consistent with classical gene conversion or homologous recombination. The exception involves a recombination/deletion event involving the ribosomal operons which occurred in the cross between parental strains RC-L2(s)/3 and RC-J/6276^tet^ yielding recombinant strain RC-J(s)/122 (Table 1, cross 12). This cross led to a deletion of one of the three ribosomal operons found in most of the in vitro-generated Tet^r^*C. trachomatis* strains (Figure 1, [5]), returning the progeny strain to the number of ribosomal operons found in wild-type *C. trachomatis* and other closely related species (Figure 4). This event also led to the deletion of the *C. trachomatis* ORFs CT740-749, resulting in a progeny strain that contains only the *C. suis* homologs of CT740 through CT749. The results demonstrate that these *C. suis* sequences can complement any required function of the deleted *C. trachomatis* genes for growth in vitro.

Nucleotide sequence analysis of the recombinant genomes showed that some of these isolates lacked the chlamydial plasmid (Table 1, Figure 1). We originally hypothesized that loss of the plasmid was associated in some way with the recombination process. To explore this possibility, PCR analyses were performed on all recombinants, as well as the parents used in this study. Both the J/6276^rif^ and the F(s)/70^rif^ parents were negative for the plasmid, whereas the L2-434^ofl^ parent was plasmid-positive (Table 1, Figure 1). Because plasmid was absent in both the J/6276^rif^ and the F(s)/70^rif^ parents used in the crosses, plasmid loss in the resulting progeny was likely a function of stress associated with antibiotic-based selection of strains prior to generating recombinants as opposed to a stress induced by the recombination process.

The sequenced recombinant genomes allowed a comparative survey of recombination events in progeny strains. The largest fragment that was laterally transferred during recombination was 412,907 base pairs, found in RC-J(s)/122, while the smallest documented double crossover event was a 7 base pair fragment in the RC-L2(s)/3 strain.

A total of 190 independent crossover regions were detected in the 12 recombinant strains. The distribution of these recombination sites was examined by mapping each crossover position from each of the 12 sequenced genomes to a single arbitrarily chosen F(s)/70 parental genome (Figure 5). There was generally a higher concentration of crossovers surrounding the *rpoB* locus (associated with Rif resistance), and there were large regions of the chromosome that lacked evidence of recombination, such as the region surrounding CT001.

A recent report [24] indicated a strong preference for recombination at specific positions within *trpB or gyrA* in several recombinant progeny originally generated by Demars and Weinfurter [4]. We used two approaches to examine selected sets of candidate hotspots identified by these authors. First, we examined our original 12 recombinant genomes for recombination events at common sites. While analysis of these fully sequenced recombinant strains identified four examples of recombination events that occurred within the same genetic region in independent progeny strains (Table 2, Figures 3 and 5), and none were found in more than 2 recombinant progeny. Second, we conducted PCR-based sequence analysis of a different set of completely independent recombinant crosses, using parental combinations (D/UW3Cx X L1/440/LN; D/UW3Cx X L3/404/LN) that were nearly identical to those analyzed by Srinivasan and colleagues [24]. Independence of these crosses was assured because each of the 14 examined progeny was the product of a fully independent cross of parental strains. In no examined case was there evidence for recombination at either of the loci identified by these authors, in any of the 14 progeny strains generated from these crosses (Table 3).

**Table 2:** A comparison of shared crossover sites in different progeny strains

**Recombinant**	**RC-L2(s)/3**	**RC-F(s)/342**
Region of crossover	CT778 (*priA)*	CT778 (*priA)*
Coordinates	916870 : 917156	954495 : 955597
Comments	F(s)70 - L2-434 hybrid CT778	F(s)70 - J/6276 hybrid CT778
Recombinant	RC-L2(s)/3	RC-J/966
Region of crossover	CT331 (*dxs*) and CT332 (*pykF)*	CT332 (*pykF)*
Coordinates	377279 : 377995	370626 : 37785
Comments	F(s)70 CT331, L2-434 CT332	J/6276 - L2-434 hybrid CT332
Recombinant	RC-L2/971	RC-J/966
Region of crossover	CT569 (*gspG*) and CT570 (*gspF*)	CT569 (*gspG*) and CT570 (*gspF*)
Coordinates	634854 : 636140	635246 : 636532
Comments	J/6276 CT569, L2-434 CT570	J/6276 CT569, L2-434 CT570
Recombinant	RC-L2/971	RC-L2/55
Region of crossover	CT585 (*trpS*) and CT586 (*uvrB*)	CT586 (*uvrB*)
Coordinates	655362 : 656561	656865 : 657292
Comments	L2-434 CT585, J/6276 CT586	F(s)70 - L2-434 hybrid CT586

**Table 3:** Analysis of independent recombinant strains for recombination hot-spots

**Strain**	**CT189 genotype**	**CT315 genotype**
L3XD_1	D	L3
L3xD_8	D	L3
L3xD_9	D	L3
L1xD_11	D	L1
L1xD_12	D	L1
L1xD_14	D	L1
L1xD_15	D	L1
L1xD_16	D	L1
L1xD_17	L1	L1
L1xD_18	D	L1
L1xD_19	D	L1
L1xD_20	D	L1
L1xD_21	L1	L1
L1xD_23	D	L1

Individual recombinant progeny from independent crosses were subjected to PCR-based DNA sequencing and assessed for recombination at positions identified as hotspots by Srinivasan and colleagues [24]. For each sequenced product, the identified genotype at that region is indicated (D or L1/L3). There were no examples of recombination in any of these sequenced PCR products.

Chi sites have previously been described as sites for homologous recombination in bacteria [25,26], and it is possible that a canonical chi site, or other sequence pattern, might be found at or near chlamydial recombination sites. The program MEME [27] was used to determine if any identified crossover sites were linked to a common sequence motif. These analyses support the hypothesis that recombination in vitro does not require specific target sequences and occurs at random sites across the genome.

### Genotypes associated with attachment efficiency

**Figure 6:**
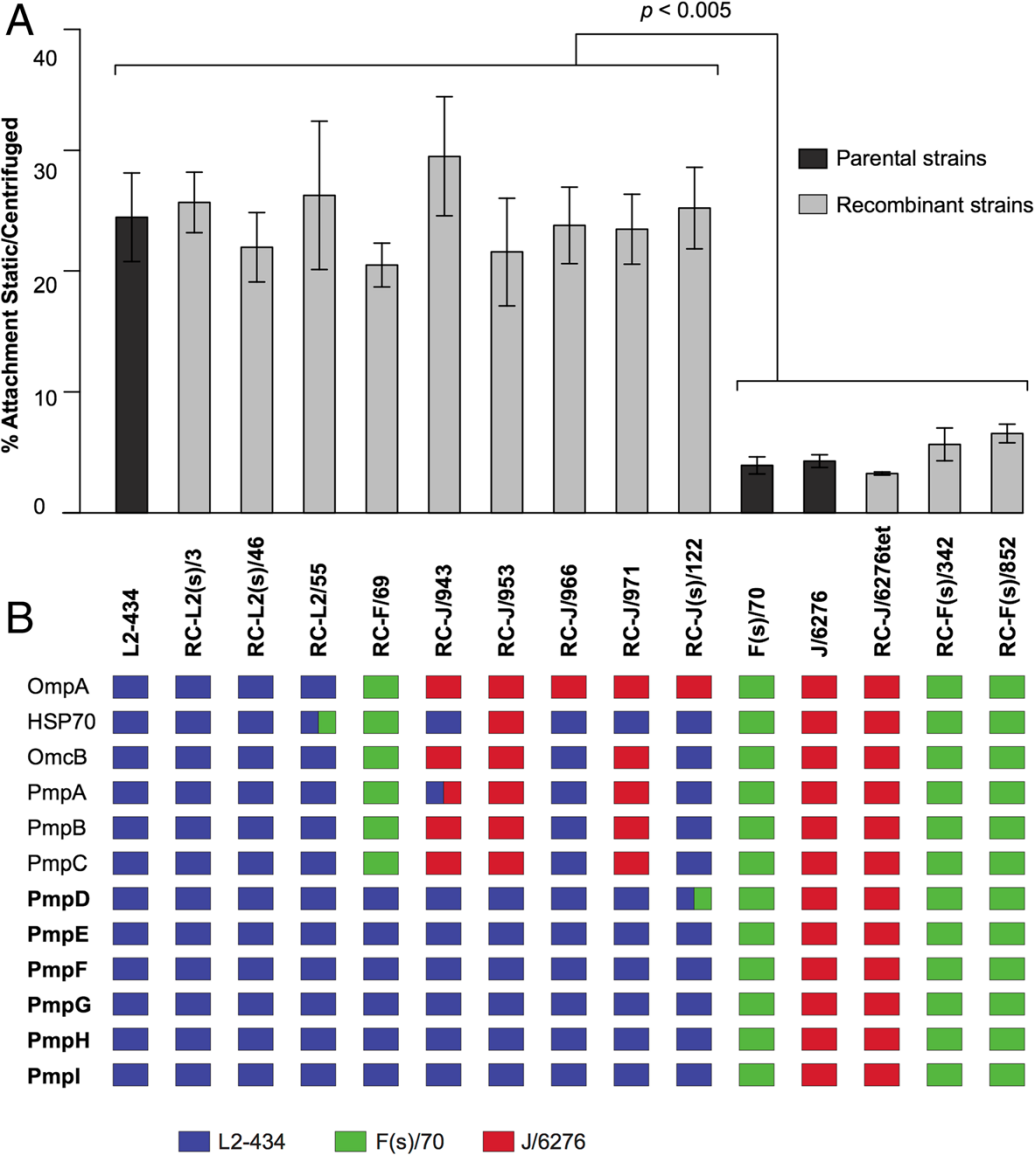
**Attachment efficiency and subsequent genomic analysis of parental and progeny recombinant strains.** Panel **A**: Measurement of the attachment efficiency for parental and recombinant strains. The specific strains analyzed are represented on the x-axis (center of figure), and the percent attachment efficiency is represented on the y-axis. Dark gray bars represent parental strains, and light gray bars represent recombinant strains. Panel **B**: The genotype of each strain for the 9 *pmp* genes and 3 other genes previously discussed as being associated with attachment are shown below each strain in graph. The colored boxes indicate the parental genotype of each gene, as indicated at the bottom of the figure. The *pmp* genes that are associated with attachment efficiency are indicated in bold. Boxes containing two colors indicate that a crossover event occurred within the gene in this strain.

Attachment efficiency in the presence or absence of centrifugation is a differentiating phenotype among *C. trachomatis* strains [22]. Strains of serovar L2 have a high rate of attachment in static culture, while the non-LGV serovars have a reduced ability to infect in the absence of centrifugation (Figure 6, [22]). We used a PCR-based analysis of attached EBs to examine the efficiency of attachment in our recombinant strains, relative to the parents of the crosses. Parental strains performed as predicted in these assays, with our serovar L2 strain having little dependence on centrifugation for attachment, while centrifugation enhanced attachment by both the serovar F and Serovar J parental strains (Figure 6). However, the different recombinant progeny strains showed variability in attachment efficiency relative to *ompA* genotype, with individual progeny strains reflecting the attachment efficiency of either the Serovar L2 or serovar F/J parental strain.

A genome-wide association analysis was then used to determine if regions in the chlamydial genome could be associated with the observed attachment efficiency phenotype. Briefly, the sequenced recombinant genomes are aligned (12 recombinant strains and 3 parental strains), and every informative site (any position in the alignment where a different genotype is present) is analyzed using the Fisher’s exact test to determine if that genotype is associated with observed phenotype. Five genomic regions were identified that had the highest possible inverse Log *p-*value based on sample size and each observation group size (Additional file 1: Figure S1). These include regions containing ORFs CT001 – CT089, CT139 – CT146, CT203 – CT215, CT740 – CT746, and CT783 – CT875. The predicted proteins encoded within these regions of the 3 parents and 12 recombinants were then compared using the MUSCLE sequence alignment software, and a total of 124 proteins had at least one non-synonymous amino acid change that was associated with the attachment phenotype (Additional file 2: Table S1). The chlamydial membrane proteins PmpE (14 amino acid changes), PmpF (110 AA changes), PmpG (28 AA changes), and PmpH (57 AA changes) were among the proteins with the highest number of non-synonymous amino acid changes. Other relevant genes that were associated with high attachment efficiency were ORFs CT089, and CT860 - 862, ORFs encoding proteins involved in the Type III secretion process [28,29]. Differences in the sequences of proteins demonstrated by others to function in primary attachment (OmpA, [30], OmcB [31]) or proposed to be associated with very early events following contact (HSP70, [32]) were not associated with differential attachment efficiency, as measured by our assay (Figure 6).

### Variation in secondary inclusion formation between recombinant strains

**Figure 7:**
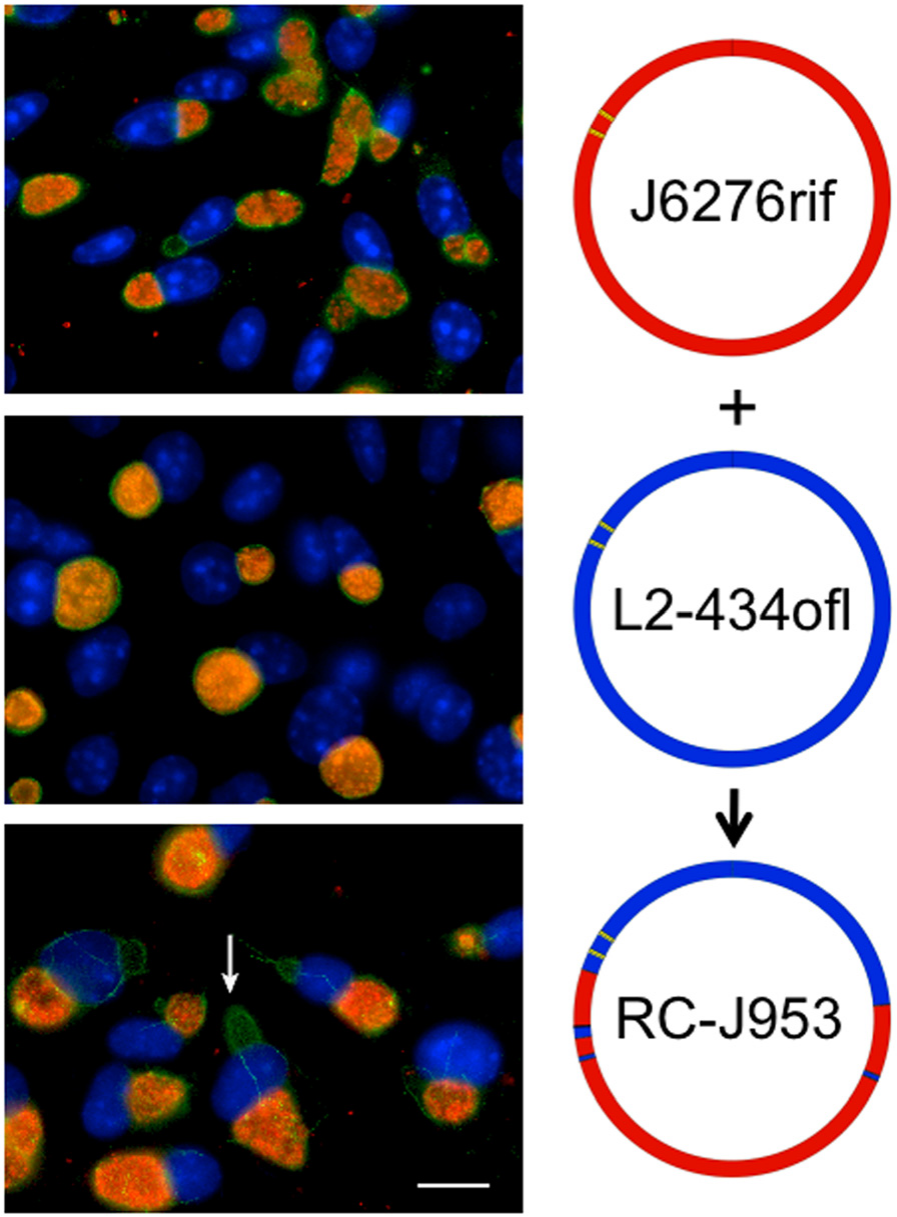
**Fluorescent microscopic analysis of the secondary inclusion formation phenotype of recombinant strain RC-J/953.** McCoy cells were infected at an MOI of ~0.5, and images were taken 48 h post-infection. All cells were labeled with anti-IncA (green), and anti-OmpA (red), and DNA is labeled with DAPI (blue). A representative secondary inclusion is indicated by the white arrow in the bottom panel. The strain being analyzed is shown at the right of each image. Scale bar, 10 μm.

Formation of secondary inclusions in infected cells is another trait that varies among strains and serovars. For example, strains of serovars G and F commonly form secondary inclusions at a higher rate than strains of serovar J and L2 [23]. We explored the secondary inclusion phenotype of IncA-positive recombinant strains; this analysis was not possible in strains that are IncA-negative, because our readout of secondary inclusions is dependent on antibodies to IncA. Of the eight IncA-positive recombinant strains tested, recombinants RC-J/953 and RC-L2/971 showed extensive secondary inclusion production (Table 1, Figure 7). These results are surprising because both parental strains (J/6276 and L2-434) used to create RC-J/953 and RC-L2/971 are low secondary inclusion formers [23]. Recombinant progeny with high secondary inclusion phenotypes where both parents exhibit low secondary inclusion formation suggest a possible interaction between at least two chlamydial proteins, or at least two independent genetic markers, in the manifestation of the secondary inclusion phenotype.

Quantitative analysis of possible loci associated with the secondary inclusion phenotype was inconclusive. This was a function of both the low number of recombinants available for analysis, and the fact the apparently multiple alleles are involved.

## Discussion

Genetic manipulation of chlamydiae has been a historical barrier to research in this system, but some of these barriers have recently been removed. One such area of progress is the use and understanding of chlamydial recombination. There is considerable evidence for in vitro and in vivo recombination by chlamydiae, and the methods for generating chlamydial recombinants are becoming routine [4,5,24]. However, there remains a general lack of understanding regarding the cellular and molecular mechanisms associated with the process. The present study was initiated to address these challenges. We hypothesized that an investigation of both the process of genetic recombination in chlamydiae and the correlation of specific chlamydial genotypes with phenotypes can be addressed using a combination of contemporary genome sequencing technologies with our ability to create genetic recombinants among chlamydiae. This approach has also been used by Nguyen and colleagues [33] as part of a forward genetic strategy in these organisms, and the results of such experiments can be integrated with the recently developed chlamydial transformation system [3] to develop and validate correlations between gene structure and protein function.

Evidence for recombination in chlamydiae was first provided by nucleotide sequencing of genes or genomes taken from a variety of chlamydial strains. There are data in the literature suggesting that recombination hotspots might be present within or around *ompA*[7,11,12], and also at other locations in the genome [34]. Our genome sequencing has added some support for this premise, as the D(s)/2923 genome discussed by Jeffrey et al. [10] has a hybrid D/E OmpA sequence, and apparent recombination sites within this strain are at or very near sites seen in other, independently isolated, clinical strains [9,11]. Other investigators have proposed and debated the concept of chlamydial recombination hotspots using analysis of chlamydial genome sequences from laboratory-generated or clinical strains [8,24,35]. In the present study, we used two strategies to investigate the possible clustering of recombination events in vitro. First, we analyzed apparent crossover sites by genome sequencing of 12 recombinant genomes, which led to the identification of a total of 190 primary recombination sites. The largest integrated fragment identified in these experiments was over 400,000 base pairs, which constitutes approximately 40% of the chlamydial genome. The long recombined region observed in these progeny strains are consistent with the original observations of Demars and Weinfurter [4], who discuss very large exchanges in their recombinants. Sequence data from clinical isolates do not provide evidence for such large exchanged fragments, but there is clear evidence of recombined regions of greater than 50,000 base pairs [6,10,35]. Chlamydial genomes are remarkably homogenous and syntenous, and we hypothesize that many recombination events that may have occurred, both in clinical strains and in laboratory-generated progeny, have been missed because of the low level of overall genomic variability.

Our data provided no evidence for increased frequency of particular recombination at specific sites surrounding markers used for selection (Figure 5). Certain areas of the genome were apparently devoid of recombination events, but these areas also were not physically linked to any of the selectable markers used for these studies. Our data provide no basis for these chromosomal sections being refractory to recombination. A total of four genomic locations were identified as possible recombination targets in more than one independent progeny clone. None of these four positions is identified as a recombination hotspot in other studies [9]. No candidate hotspot regions within or immediately around *ompA* were identified in any of our in vitro recombinants, and none of the positions are directly flanking the markers used for selection.

A second approach to investigate chlamydial recombination hotspots was in response to work of Srinivasan et al. [24] who examined sequence data generated by Demars and Weinfurter [4]*,* and identified candidate recombination hotspots at several loci. We attempted to replicate these results by making completely independent recombinant clones using strains very similar to those used by these investigators, and examining predicted loci for evidence of recombination. These clones were determined to be fully independent, because each was derived from a completely independent primary mixture of parental strains. We found no evidence of the use of recombination sites identified by Srinivasan and colleagues in any of the clones. Our inability to identify any hotspots surrounding previously identified recombination sites leads us to propose that most previously identified recombination hotspots were identified as such because: 1) there was significant in vivo selection pressure for change at a locus (i.e. intra-OmpA or Pmp antigenic variation), or 2) the position being analyzed is identified because there simply was more sequence heterogeneity in that region of the chromosome, or 3) the in vitro progeny identified as containing recombination hotspots were siblings in a single recombination event prior to being cloned out of a population.

Each recombination event identified appeared to be a product of homologous recombination or gene conversion between highly related sequences. There was a single deletion event in one progeny strain, in which two virtually identical rRNA sequences were precisely deleted to yield a single rRNA operon, with 17 kB of intervening sequences (10 genes, CT740 through CT749) removed in the process [RC-J(s)/122, Figure 4]. This was the only example of a deletion in any progeny strain, and there were no cases of a duplication event. These results are consistent with the general sequence similarity and synteny found in the naturally mosaic *C. trachomatis* genomes, with occasional phenotype-associated deletions in specific regions of the chromosome [36,37].

The three-way crosses were designed to examine the possibility that multiple parents could be involved in generation of a single recombinant progeny. We saw no evidence of a three-way cross in any of our selection experiments or in any genome sequence analysis, even though multiple independent two-way crosses were recovered from those experiments. If the probability of a three-way event is a function of the probability of two independent recombination events, it is likely that not enough individual recombinants were screened to identify an extremely rare progeny clone. There is, however, one issue that is addressed by the absence of any evidence for contribution of three parents in a cross. In many of the recombinants identified by our group and in studies by Demars and colleagues [4,38], multiple fragments from each parental genome are found in a recombinant progeny, often in regions of the chromosome that were not selected for with the tested antibiotics (Figures 3 and 5). It is possible that these differently recombined fragments involve sequential and independent recombination events occurring during the mixed infections used in this procedure. If involvement of multiple chlamydiae was a common occurrence in the generation of a cross, we hypothesized that some progeny from the three-way crosses should carry fragments of each parent. As no single progeny strain was identified with fragments of each of the parents in the three-way cross, our results do not support this hypothesis. Therefore our current model is that the generation of recombinant progeny is the result of a single exchange event between two parents, and that these exchanges can involve very large fragments of the chromosomal DNA. This latter result is consistent with analyses by other laboratories [4,9,33,35,38]. Subsequent recombination events will then lead to differential integration of fragments of the exchanged DNA, leading to the mosaicism seen in many of the recombinants.

The attachment efficiency in the absence of centrifugation measured for the different recombinants revealed groups having either a high attachment efficiency, as exhibited by LGV strains, or a low attachment efficiency, as exhibited by non-LGV urogenital strains (Figure 6). Genome wide association analysis of this phenotype revealed a number of loci that were quantitatively associated with the attachment efficiency phenotype seen in cell culture. While the list of candidate alleles that might be associated with this phenotype includes a wide variety of genes (i.e. type III secretion –associated ORFs [28,29]), we focus this discussion on proteins known or hypothesized to be on the surface of the chlamydial elementary body. One collection of proteins that is highly associated with attachment efficiency were six of the Pmp proteins (PmpDEFGHI), a group of chlamydia-specific autotransporters, some of which are known to be surface exposed and are logical mediators of attachment to cells [39]. A bioinformatic analysis of Pmp sequence and structure demonstrates that four of these encoded Pmps (PmpEFGH) vary consistently in relationship to the described phenotype, and these changes include alterations of the net negative charge of the Pmp protein (Additional file 2: Tables S1-S2 and Additional file 3: Table S2). It is, however, preliminary to assess any property of a single protein or small set of proteins with the attachment efficiency distinction among strains. The recently developed genetic transformation system will be a critical technology in directly assessing such relationships in this species [3].

The second phenotype investigated in our study was the formation of secondary inclusions within infected cells. This property of *C. trachomatis* strains varies not only between *C. trachomatis* serovars, but also between strains within serovars [23]. An intriguing result was the identification of high secondary inclusion formers in crosses between parents that exhibited very low secondary inclusion formation phenotypes (Table 1, Figure 7). While interpretations of this result are preliminary, it appears that the phenotype is associated with two or more regions of the genome, and that a specific combination of genotypes at these positions is required for the high secondary inclusion formation phenotype to be manifested.

Continued examination of novel recombinants, including backcrosses to integrate more parental genome into recombinant strains will add clarity to the phenotypes we have discussed. We also continue to use the recombinants as tools to understand the basic processes associated with genetic exchange in the chlamydiae.

## Conclusion

The described experiments characterize in detail the products of genetic exchange by *C. trachomatis* in vitro. Sequences representing over 1/3 of the chlamydial chromosome can be incorporated during these crosses. Selected phenotypes can be segregated in these crosses. This approach can be combined with the novel DNA transformation technologies being developed in these bacteria, leading to novel approaches for determining the relationship between genetic makeup and chlamydial phenotype, both in vitro and in vivo.

## Methods

### Chlamydial strains and selection for resistance

Antibiotic resistant *C. trachomatis* strains J/6276^rif^, RC-J/6276^tet-rif^, F(s)/70^rif^, F(s)/70^tet-rif^ L2-434^ofl^,DUW/3Cx ^ofl^, L1/440/LN^rif^ or L3/404/LN^rif^ were generated as previously described [5]. Briefly, strains were grown in McCoy cells at a multiplicity of infection (MOI) of 1 in media containing sub-inhibitory concentrations, equivalent to half the minimum inhibitory concentration (MIC) of the appropriate drug. Serial passages of these strains were cultured in the media containing desired antibiotics until resistant mutants emerged or until passage was completely negative. Some strains required several attempts until resistant mutants were isolated. Isolates were then cloned by a twofold limiting dilution method. The resulting cloned elementary bodies (EBs) were grown to high titers and were partially purified by centrifugation of lysates of infected cells through a 30% MD-Gastroview® pad (Mallinckrodt Inc. St Louis).

### Generation of recombinant clones for complete genome sequence analysis

Recombinants isolated for genome analysis were generated from two sets of crosses (Table 1). The first of these involved two parental strains; L2-434^ofl^ and F(s)/70^rif^ and the second was a three-way cross with the parental strains F(s)/70^tet-rif^, J/6276^rif^ and L2-434^ofl^. Recombination experiments were conducted as previously described [5]. Briefly, crosses were performed in McCoy cells seeded in sets of individual shell vials. The monolayers were then infected with different combinations of drug-resistant strains each at an MOI = 2, ensuring infections of cells with both strains. Cultures were incubated for 48 h post-infection in the absence of antibiotics and were then detached and lysed using a -80C/37C freeze-thaw cycle [5]. Potential recombinants were selected by inoculating 50 μl of the freeze-thaw lysates from each shell vial onto a new shell vial monolayer and overlaying with a medium containing antibiotics at 1/4 the MIC for each resistant parental strain. In the case of the three-way cross [F(s), J, L2], three different combinations of drug were applied to the infected monolayers (MOI = 2). These combinations included ofloxacin/rifampicin, ofloxacin/tetracycline, and ofloxacin/rifampicin/tetracycline.

### Generation of recombinant chlamydial strains for analysis of recombination hot spots

Multiple independent shell vials containing confluent McCoy cells were inoculated sequentially with ofloxacin-resistant D/UW3Cx and rifampin-resistant L1/440/LN or L3/404/LN strains, and incubated 48 h in medium lacking antibiotics. Monolayers were lysed and used as inocula onto fresh McCoy cells at MOI = 1, and incubated in the presence of 4X the MIC of the drugs used for selection, rifampin and ofloxacin. These concentrations were previously determined to be sufficient to select for individual recombinant strains resistant to both drugs. Incubation of either parent in this combination and concentration of antibiotics at MOI = 1 never yielded a doubly resistant mutant parent. Chlamydial recombinants growing in this mixture of antibiotics were propagated and cloned by limiting dilution. Only a single recombinant progeny was collected from each lineage from a single original inoculated shell vial. DNA was harvested from these clones, and PCR primers were created that flanked regions of suspected recombination hotspots identified by Srinivasan and colleagues [24]. The Phusion high fidelity DNA polymerase (New England Biolabs, Ipswich, MA) was used to generate PCR products from these regions, and these were sequenced at the Oregon State University Center for Genomics Research and Biocomputing. Sequences were then examined for possible recombination by comparing informative sequence polymorphisms relative to each parent in the cross.

### Chlamydia recombinant strain genomic DNA preparation

Recombinants were clonally isolated using limiting dilution and EB purification was conducted as previously described [23,40]. Purified EBs were incubated for 60 min with 4 units/mL RQ1 DNase (Promega) followed by treatment with 2 mM EGTA (RQ1 Stop solution, Promega) to inactivate the DNase. Elementary bodies were then suspended in Qiagen Genomic buffer B1 supplemented with dithiothreitol (5 mM) and DNA was then extracted using the Qiagen Genomic Tip kit, (Qiagen, Valencia, CA) following the manufacturer’s instructions.

### Genome sequencing and sequence analysis

Genomic DNA from recombinant strains was processed for Illumina-based paired-end sequencing using commercial DNA preparation kits (Illumina Inc., San Diego, CA) following the manufacturer’s instructions. Each recombinant genome was first assembled using the reference-guided assembly program Maq [41]. Appropriate parental genomes were used as references in the analyses. Regions in reference-guided assembled genomes where Maq could not resolve sequence were then compared to contiguous sequences assembled using de-novo assembly software Velvet [42] and a single contiguous draft sequence was produced.

To confirm the clonality of the recombinant genomes, and to quality control our assembly process, two to four apparent crossover regions in each recombinant progeny were amplified by PCR and sequenced using classical Sanger sequencing. In all cases the sequenced amplicon contained the appropriate informative sites from each parent involved in the cross (not shown).

Recombinant maps of each genome were produced by computationally parsing a draft genome against the two parents used to generate the recombinant, using the alignment program MAFFT with the default settings [43,44]. Any detected crossover regions were manually analyzed using MacVector sequence analysis software (Cary, NC). Crossover regions were defined as the intervening homologous sequence between two informative sites (defined as a nucleotide position that varied in sequence between the two parent genomes), where the informative site was the same as one parent at one position and the same as the second parent at an immediately adjacent informative site.

Whole genome alignments including all recombinant strains and the 3 parental strains were constructed using MAFFT with default settings. Any position in this alignment where at least one genome had a variable base was further analyzed using the Fisher exact test as a metric to determine if the variable genotype could be associated with a given phenotype. In these analyses, a low *p*-value indicated an association between the base sequence and a specific parental phenotype or genotype. A variable genotype was considered to be associated with a given phenotype if the calculated *p-*value was the lowest possible based on the sample size. The *p-*values calculated by the Fisher’s exact test were inverse Log-transformed and plotted using the statistical analysis program R (http://www.r-project.org/).

Whole genome alignments of all recombinants against both parents were used to determine if any random mutations had occurred during culture and the generation of recombinants. A random mutation was defined as a nucleotide in the recombinant sequence that was different than the nucleotide of either parent at the same nucleotide position. All ORF designations are based on numbering system used for the *C. trachomatis* D/UW3 genome sequence [31].

### Measurement of attachment efficiency

McCoy cell monolayers were seeded in duplicate 24 well plates at 90% confluency, and triplicate wells of each plate were infected using a target MOI = of 1. Plates were then either centrifuged at 640 × g (2000 RPM on Beckman Coulter, Allegra X-15R centrifuge) for 1 h or placed on a rocker platform for 1 h, with both treatments being at room temperature. Wells were then washed 3 times with Hanks balanced salt solution and DNA was extracted directly from each well using the Qiagen DNeasy Blood and Tissue kit, with the lysis buffer supplemented with 5 mM dithiothreitol. Each sample was pipetted up and down 10 times to disrupt both host cells and chlamydiae. Genome copy number was determined for each treatment by qPCR, using a probe for hsp60 (groEL_3, CT755). A cloned and quantified version of CT755 was used as the standard curve on all qPCR plates, ensuring that each sample being analyzed was properly quantified. The target sequence for this assay is conserved among *C. trachomatis*, but was unique to this hsp60 allele, as demonstrated by PCR analysis of alternate hsp60 open reading frames (CT110 and CT604; not shown). Attachment efficiency was then calculated by dividing the genome copy number of the rocked samples by the genome copy number of the centrifuged samples.

### Quantification of secondary inclusion formation

The frequency of secondary inclusion formation in parental and progeny strains was determined using previously described methods [23]. Briefly, McCoy cells were infected with the strain of interest at an MOI = of 0.3. These cells were incubated for approximately 24 hpi prior to fixation with methanol. *C. trachomatis* IncA was labeled with mouse monoclonal anti-IncA, and chlamydial developmental forms were labeled with mouse anti-lipopolysaccharide [23]. Cells were then labeled with appropriate secondary antibodies (Southern Biotechnology Associates, Birmingham, AL) and observed using 400× or 1000× magnification. A semi-quantitative measure of secondary inclusion formation was conducted by determining the fraction of infected cells having secondary inclusions versus the total number of infected cells. A 1+ to 4+ scoring system was used to quantify secondary inclusion formation and each score was determined on three independent sets of coverslips. A 1+ value is equivalent to less than 10% of infected cells having secondary inclusions, while a 4+ value is equivalent to 40% or more of the cells having secondary inclusions.

### Genome sequence accession numbers

The genome sequences of the parental strains used to generate recombinant sequences and the previously sequenced *C. trachomatis* strains used in the whole genome alignment studies are in the DDBJ/EMBL/GenBank database under the following accession numbers: D/UW3Cx, AE001273; L2-434Bu, AM884176; L2/UCH1, AM884177; L1/440/LN, HE601950; L3/404/LN, HE601955; D(s)/2923, ACFJ01000001; E/11023, CP001890; E/150, CP001886; G/9768, CP001887; G/11074, CP001889; G/11222, CP001888; F/70, ABYF01000001; F(s)/70, ABYG01000001; J/6276, ABYD01000001; J(s)/6276, ABYE01000001.

The *C. trachomatis* genome accession numbers of the recombinants used in this study have been deposited in the DDBJ/EMBL/GenBank database under the following accession numbers: RC-F/69, CP002671; RC-L2(s)/46, CP002672; RC-F(s)/852, CP002673; RC-J/943, CP002674; RC-J/953, CP002675; RC-L2(s)/3, CP002676; RC-F(s)/342, CP002677; RC-J(s)/122, CP002678; RC-J/966, CP002679; J/6276tet1, CP002680; RC-L2/971, CP002681; RC-L2/55, CP002682.

## Competing interests

The authors declare they have no competing interests.

## Authors’ contributions

BJ sequenced and assembled genomes, performed comparative genomics, and conducted the attachment assays with the help of SE. RS generated all recombinant strains and scored for secondary inclusion phenotype. KS contributed to study design and data analysis. DR was responsible for overall study design and data analysis. BJ, RS, and DR drafted the manuscript. All authors read and approved the final manuscript.

## Supplementary Material

Additional file 1: Figure S1 Genome-wide association analysis of the attachment efficiency phenotype. Genome-wide *p-*values from Fisher’s exact test are given on the Y-axis. The results were collected from an alignment of the twelve recombinants and the three parents used for creating the recombinants. Genome position is indicated along X-axis, beginning with CT001 as defined for the DUW/3 genome [31]. The brackets and ORF numbers indicate the genes present in the genomic regions showing the highest inverse *p*-values in these analyses. Click here for file

Additional file 2: Table S1 Gene products associated with attachment efficiency phenotype. D/UW3 and L2-434 gene designations, and putative membrane localization are given for gene products with amino acid changes that are associated with attachment efficiency. NS AA changes indicate the number of non-synonymous amino acid changes that are associated with attachment efficiency. Indel status indicates whether an in-frame insertion or deletion within a protein is associated with attachment efficiency. Elongation/truncation status indicated whether a protein has either an N or C-terminal truncation/elongation that is associated with attachment efficiency. Click here for file

Additional file 3: Table S2 Polymorphic membrane protein charge analysis. The numbers below each of the Pmp is the charge of the protein at a pH of 7. The results of the three parental strains used in this study as well as three previously sequenced non-LGV urogenital strains are shown. Click here for file
