# Supplementary figures and images for: Genomic and phenotypic characterization of in vitro-generated Chlamydia trachomatis recombinants
Source: BMC Microbiol. 2013 Jun 20;13:142. doi: 10.1186/1471-2180-13-142 (PMC3703283; doi:10.1186/1471-2180-13-142)

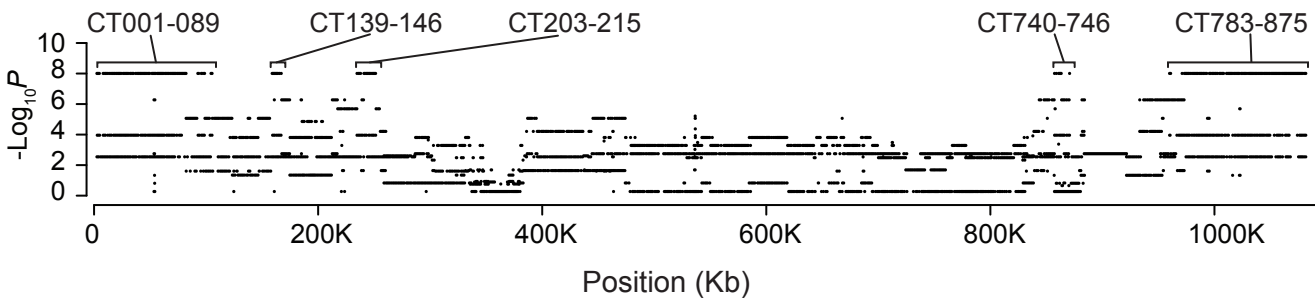

Supplement: Additional file 1: Figure S1 — Genome-wide association analysis of the attachment efficiency phenotype. Genome-wide p-values from Fisher’s exact test are given on the Y-axis. The results were collected from an alignment of the twelve recombinants and the three parents used for creating the recombinants. Genome position is indicated along X-axis, beginning with CT001 as defined for the DUW/3 genome [31]. The brackets and ORF numbers indicate the genes present in the genomic regions showing the highest inverse p-values in these analyses. [file 1471-2180-13-142-S1.pdf]
