# Supplementary material for: Genomic and phenotypic characterization of in vitro-generated Chlamydia trachomatis recombinants
Source: BMC Microbiol. 2013 Jun 20;13:142. doi: 10.1186/1471-2180-13-142 (PMC3703283; doi:10.1186/1471-2180-13-142)
Supplement: Additional file 2: Table S1 — Gene products associated with attachment efficiency phenotype. D/UW3 and L2-434 gene designations, and putative membrane localization are given for gene products with amino acid changes that are associated with attachment efficiency. NS AA changes indicate the number of non-synonymous amino acid changes that are associated with attachment efficiency. Indel status indicates whether an in-frame insertion or deletion within a protein is associated with attachment efficiency. Elongation/truncation status indicated whether a protein has either an N or C-terminal truncation/elongation that is associated with attachment efficiency. [file 1471-2180-13-142-S2.pdf]

D/UW3 locus	L2-434 locus	Gene product	NS AA changes	Membrane protein	Indels	Elongation/truncation
CT001	CTL0256	hypothetical protein	2	putative membrane protein		
CT005	CTL0260	hypothetical protein	1	putative membrane protein		
CT007	CTL0262	hypothetical protein	2	putative membrane protein		
CT010	CTL0265	htrB	1			
CT011	CTL0266	hypothetical protein	2			
CT012	CTL0267	hypothetical protein	1	putative membrane protein		
CT013	CTL0268	cydA	4	putative membrane protein		
CT014	CTL0269	cydB	3	putative membrane protein		
CT015	CTL0270	hypothetical protein	1			
CT016	CTL0271	hypothetical protein	2			
CT017	CTL0272	hypothetical protein	3	putative membrane protein		
CT018	CTL0273	hypothetical protein	6	putative membrane protein		
CT019	CTL0274	ileS	5			
CT021	CTL0276	hypothetical protein	1	putative membrane protein		
CT022	CTL0277	rpmE	1			
CT025	CTL0280	ffh	1			
CT027	CTL0282	trmD	1			
CT032	CTL0287	metG	2			
CT034	CTL0289	hypothetical protein	3	putative membrane protein		
CT035	CTL0290	hypothetical protein	1			
CT036	CTL0291	hypothetical protein	20	putative membrane protein	yes	yes
CT040	CTL0296	ruvB	1			
CT041	CTL0297	hypothetical protein	1			
CT042	CTL0298	hypothetical protein	3			
CT045	CTL0301	hypothetical protein	2			
CT046	CTL0302	hct2	5			
CT047	CTL0303	hypothetical protein	1			
CT049	CTL0305	hypothetical protein	30			
CT050	CTL0306	hypothetical protein	19	putative membrane protein	yes	
CT051	CTL0307	hypothetical protein	63	putative membrane protein	yes	
CT052	CTL0308	hemN	1			
CT054	CTL0310	sucA	6			
CT056	CTL0312	hypothetical protein	1			
CT057	CTL0313	gcpE	3			
CT058	CTL0314	hypothetical protein	11	putative membrane protein	yes	
CT059	CTL0315	fer	2			
CT060	CTL0316	flhA	4	putative membrane protein		
CT061	CTL0317	fliA	1			
CT062	CTL0318	tyrS	1			
CT063	CTL0319	gnd	4			
CT065	CTL0321	hypothetical protein	1	putative membrane protein		
CT076	CTL0332	smpB	1			
CT079	CTL0335	hypothetical protein	1	putative membrane protein		
CT082	CTL0338	hypothetical protein	3	putative membrane protein		
CT083	CTL0338A	hypothetical protein	2			
CT087	CTL0342	malQ	1			
CT089	CTL0344	copN	5	putative membrane protein		
CT140	CTL0395	hypothetical protein	3	putative membrane protein		
CT142	CTL0397	hypothetical protein	8	putative membrane protein		
CT143	CTL0398	hypothetical protein	8			
CT144	CTL0399	hypothetical protein	20	putative membrane protein	yes	
CT146	CTL0401	dnlJ	2			
CT205	CTL0457	pfkA	2			
CT206	CTL0458	hypothetical protein	1			
CT207	CTL0459	pfkA_2	2			
CT208	CTL0460	gseA	2	putative membrane protein		
CT209	CTL0461	leuS	5			
CT210	CTL0462	hemL	4			

D/UW3 locus	L2-434 locus	Gene product	NS AA changes	Membrane protein	Indels	Elongation/truncation
CT211	CTL0463	hypothetical protein	1			
CT212	CTL0464	hypothetical protein	2			
CT214	CTL0466	hypothetical protein	5	putative membrane protein		
CT215	CTL0467	dhnA	3			
CT783	CTL0152	hypothetical protein	2			
CT784	CTL0153	rnpA	1			
CT795	CTL0164	hypothetical protein	1			
CT796	CTL0165	glyQ	2			
CT798	CTL0167	glgA	2			
CT799	CTL0168	rplY	1			
CT804	CTL0173	ispE	1			
CT806	CTL0175	ptr	2	putative membrane protein	yes	
CT807	CTL0176	plsB	1		yes	
CT808	CTL0177	cafE	1			
CT809	CTL0178	hypothetical protein	7		yes	yes
CT812	CTL0183	pmpD	2	membrane protein	yes	
CT813	CTL0184	hypothetical protein	12	putative membrane protein		
CT815	CTL0187	hypothetical protein	3			
CT816	CTL0188	glmS	4			
CT817	CTL0189	tyrP	2	putative membrane protein		
CT818	CTL0190	hypothetical protein	2	putative membrane protein		
CT820	CTL0192	ftsY	1			
CT821	CTL0193	sucC	4	putative membrane protein		
CT823	CTL0195	htrA	3			
CT824	CTL0196	hypothetical protein	4			
CT825	CTL0197	rmuC	4	putative membrane protein		
CT826	CTL0198	pssA	1	putative membrane protein		
CT827	CTL0199	nrdA	7			
CT828	CTL0200	nrdB	1	putative membrane protein		
CT830	CTL0202	hypothetical protein	2			
CT831	CTL0203	murB	1			
CT832	CTL0204	nusB	1			
CT833	CTL0205	infC	38		yes	yes
CT836	CTL0208	pheS	1			
CT837	CTL0209	hypothetical protein	6			
CT839	CTL0211	hypothetical protein	1	putative membrane protein		
CT840	CTL0212	tilS	1			
CT841	CTL0213	ftsH	3	putative membrane protein		
CT842	CTL0214	pnp	4			
CT846	CTL0218	hypothetical protein	1	putative membrane protein		
CT847	CTL0219	hypothetical protein	2			
CT848	CTL0220	hypothetical protein	3			
CT850	CTL0223	hypothetical protein	2	putative membrane protein		
CT852	CTL0225	hypothetical protein	12	putative membrane protein	yes	yes
CT853	CTL0226	hypothetical protein	1	putative membrane protein		
CT854	CTL0227	hypothetical protein	5	putative membrane protein		
CT856	CTL0231	hypothetical protein	3	putative membrane protein		
CT857	CTL0232	hypothetical protein	1	putative membrane protein		
CT858	CTL0233	cpa	1			
CT859	CTL0234	ispH	3			
CT860	CTL0235	copD2	6			
CT861	CTL0236	copB2	11	putative membrane protein		
CT862	CTL0237	lcrH	2			
CT863	CTL0238	hypothetical protein	2			
CT864	CTL0243	xerD	1			
CT865	CTL0244	hypothetical protein	2			
CT866	CTL0245	glgB	4			
CT867	CTL0246	hypothetical protein	9	putative membrane protein		
CT868	CTL0247	hypothetical protein	36	putative membrane protein	yes	yes
CT869	CTL0248	pmpE	14	membrane protein	yes	
CT870	CTL0249	pmpF	110	membrane protein	yes	
CT871	CTL0250	pmpG	28	membrane protein		
CT872	CTL0251	pmpH	57	membrane protein	yes	
CT873	CTL0252	hypothetical protein	72		yes	yes
CT874	CTL0254	pmpI	3			
CT875	CTL0255	hypothetical protein	28			

TABLE S1. Gene products associated with attachment efficiency phenotype. D/UW3 and L2-434 gene designations, and putative membrane localization are given for gene products with amino acid changes that are associated with attachment efficiency. NS AA changes indicate the number of non-synonymous amino acid changes that are associated with attachment efficiency. Indel status indicates whether an in-frame insertion or deletion within a protein is associated with attachment efficiency. Elongation/truncation status indicated whether a protein has either an N or C-terminal truncation/elongation that is associated with attachment efficiency.
