# Supplementary material for: Genomic and phenotypic characterization of in vitro-generated Chlamydia trachomatis recombinants
Source: BMC Microbiol. 2013 Jun 20;13:142. doi: 10.1186/1471-2180-13-142 (PMC3703283; doi:10.1186/1471-2180-13-142)
Supplement: Additional file 3: Table S2 — Polymorphic membrane protein charge analysis. The numbers below each of the Pmp is the charge of the protein at a pH of 7. The results of the three parental strains used in this study as well as three previously sequenced non-LGV urogenital strains are shown. [file 1471-2180-13-142-S3.docx]

TABLE S3. Polymorphic membrane protein charge analysis. The numbers below each of the Pmp is the charge of the protein at a pH of 7. The results of the three parental strains used in this study as well as three previously sequenced non-LGV urogenital strains are shown.

|  | Associated with attachment | | | | |  |  | Not associated with attachment | | |
| --- | --- | --- | --- | --- | --- | --- | --- | --- | --- | --- |
|  | **pmpD** | **pmpE** | **pmpF** | **pmpG** | **pmpH** | **pmpI** |  | **pmpA** | **pmpB** | **pmpC** |
| **L2-434** | -51.5 | -4.5 | 4.6 | -13 | -8.6 | -11.6 |  | 10.9 | 5.8 | -89.4 |
| **J/6276** | -54.5 | -7.5 | 0.6 | -22 | -16.8 | -12.4 |  | 10.9 | -20.9 | -98.7 |
| **F(s)/70** | -53.8 | -9.5 | 0.6 | -22 | -15.8 | -11.6 |  | 10.9 | -24 | -102.4 |
| **D/UW3** | -53.5 | -7.5 | 0.6 | -20.8 | -16.8 | -11.6 |  | 10.9 | -23.9 | -100.4 |
| **E/11023** | -53.8 | -7.5 | 0.6 | -20.8 | -16.8 | -11.6 |  | 10.9 | -24 | -102.4 |
| **G/9768** | -54.5 | -7.2 | -2.4 | -20.8 | -15.8 | -11.6 |  | 10.9 | -20.9 | -98.7 |
